# The Dysregulation of Eicosanoids and Bile Acids Correlates with Impaired Kidney Function and Renal Fibrosis in Chronic Renal Failure

**DOI:** 10.3390/metabo11020127

**Published:** 2021-02-23

**Authors:** Yan-Ni Wang, He-He Hu, Dan-Dan Zhang, Xia-Qing Wu, Jian-Ling Liu, Yan Guo, Hua Miao, Ying-Yong Zhao

**Affiliations:** 1 Faculty of Life Science & Medicine, Northwest University, No. 229 Taibai North Road, Xi’an 710069, China; wynxts@126.com (Y.-N.W.); huhehenwu@163.com (H.-H.H.); ddzhangsmile@163.com (D.-D.Z.); wxq-607697@163.com (X.-Q.W.); ljl2003ljl@126.com (J.-L.L.); 2 Department of Internal Medicine, University of New Mexico, 1700 Lomas Blvd NE, Albuquerque, NM 87131, USA; YaGuo@salud.unm.edu

**Keywords:** chronic renal failure, lipidomics, lipid metabolism, leukotriene metabolism, bile acid metabolism

## Abstract

Chronic renal failure (CRF) is an irreversible deterioration of the renal functions that characterized by fluid electrolyte unbalance and metabolic-endocrine dysfunctions. Increasing evidence demonstrated that metabolic disturbances, especially dyslipidemia and profound changes in lipid and lipoprotein metabolism were involved in CRF. Identification of lipids associated with impaired kidney functions may play important roles in the understanding of biochemical mechanism and CRF treatment. Ultra-performance liquid chromatography coupled with high-definition mass spectrometry-based lipidomics was performed to identify important differential lipids in adenine-induced CRF rats and investigate the undergoing anti-fibrotic mechanism of *Polyporus umbellatus* (PPU) and ergone (ERG). Linear correlation analysis was performed between lipid species intensities and creatinine levels in serum. Adenine-induced rats exhibited declining kidney function and renal fibrosis. Compared with control rats, a panel of lipid species was identified in the serum of CRF rats. Our further study demonstrated that eight lipids, including leukotrienes and bile acids, presented a strong linear correlation with serum creatinine levels. In addition, receiver operating characteristics analysis showed that eight lipids exhibited excellent area under the curve for differentiating CRF from control rats, with high sensitivity and specificity. The aberrant changes of clinical biochemistry data and dysregulation of eight lipids could be significantly improved by the administration of PPU and ergone. In conclusion, CRF might be associated with the disturbance of leukotriene metabolism, bile acid metabolism and lysophospholipid metabolism. The levels of eicosanoids and bile acids could be used for indicating kidney function impairment in CRF. PPU could improve renal functions and either fully or partially reversed the levels of eicosanoids and bile acids.

## 1. Introduction

A complex clinical syndrome, chronic renal failure (CRF), represents the end stage of various kidney diseases that characterized by renal function degradation, endocrine imbalance, and metabolic disorders associated with progressive renal injury [1]. In recent years, CRF has been considered as one of the most significant public health problems, which has become a heavy financial burden on global and local economics facing the public healthcare systems. Progression of CRF is associated with interstitial fibrosis, which is characterized by monocyte and macrophage infiltration, tubular atrophy, and fibroblast proliferation/differentiation, events that finally cause extracellular matrix accumulation and tubulointerstitial fibrosis [2,3]. Adenine is a nitrogen heterocycles compound, and adenine treatment would lead to serious renal pathological damages and precipitation in renal tubules and further contribute to accumulation of serum creatinine and blood urea nitrogen [4,5]. Therefore, an adenine-induced rats model provides a potential platform to study the progression of CRF. In the past few decades, substantial advances have been made in understanding the underlying pathophysiology of CRF, leading to the development of novel pharmaceuticals and interventional therapies [6,7]. Tragically, efficient treatment in the clinical setting are scarce and often ineffective to either stop or reverse progression of CRF, underscoring the importance of novel therapeutics on disease intervention.

In recent years, taking advantage of the novel technological advances in high-resolution mass spectrometry, lipidomics is a platform for identifying a large array of endogenous lipidome that directly reflect the biological events in the test samples, which has attracted increasing interest in the realm of life sciences and clinical diagnostics [8,9]. Lipids are crucial mediators of the human metabolism with substantial complexity and diversity, and the dysregulation of lipid metabolism is responsible for a wide range of metabolic diseases and involves in progression of acute and chronic diseases, such as chronic kidney diseases (CKD), cardiovascular diseases, and cancer, as well as patient wellbeing; thus, some dysregulated lipids may act as important biomarkers [10,11,12,13]. The progression of CRF and its complications result in marked abnormal lipid metabolism and changed serum lipid profile [14,15], whereas metabolic disturbances, including dyslipidemia and profound changes in lipid, have been frequently demonstrated that contribute to the pathogenesis and prognosis of CRF and various complications [16]. Moreover, alteration of metabolomic in kidney diseases may provide clues understanding the pathophysiology of various renal diseases development and progression [17,18]. Therefore, sensitive and specific differential lipid species may yield therapeutic insights for diagnosis and monitoring of disease progression [19]. Ultra-performance liquid chromatography coupled with high-definition mass spectrometry (UPLC-HDMS)-based lipidomics has recently been applied in the identification of important differential lipid species for progression of CRF and a variety of lipid species have been identified in progression of CKD [20,21,22,23]. Interestingly, adenine triggers an imbalance between lipid synthesis and degradation, and adenine-induced CRF rats cause metabolic abnormalities similar to kidney disease clinical symptoms in human [4]. Several causes of kidney diseases-associated dysmetabolism have been identified by our group and others studies, including inflammation, fibrotic responses, programmed cell death and oxidative stress, and regulation of nuclear factor kappa B and transforming growth factor-β (TGF-β)/Smad signaling pathways [24,25,26,27]. We believe that understanding of CRF-associated lipid metabolic perturbation could improve diagnosis and management of CRF by providing stage-specific and injury detecting important differential lipid species.

Natural products have recently become increasingly recognized as alternative therapy for the treatment of various diseases, including but not limited to the prevention and treatment of CKD [28,29,30,31]. In recent years, a myriad of natural products has been reported to exhibit beneficial effects against renal fibrosis and prevent or delay CRF by mediating lipid metabolism disorders and preserving kidney function [20,32]. *Polyporus umbellatus* (PPU) is a well-known precious medicinal fungus that has various biological activities and medicinal perspective [33,34,35,36]. Ergosta-4,6,8(14),22-tetraen-3-one (ergone, ERG) is one of the bioactive steroids of PPU and has been proven to ameliorate renal injury and renal fibrosis [37,38]. Here, we hypothesis that n-hexane extracts of PPU and ergone could modulate lipid metabolic profiling in renal fibrosis, to exert its renoprotective effect.

In the present study, UPLC-HDMS-based lipidomics combined with pattern recognition methods were performed to identify important differential lipids in adenine-induced CRF rats. Important differential lipids were chosen by analyzing correlations between serum lipid species intensities and creatinine levels using linear correlation analysis. In order to further demonstrate whether restoring lipid levels were associated with improving kidney function, we further investigate the effects of PPU and its bioactive component ergone on important differential lipids in adenine-induced CRF rats. Our findings demonstrated that the dysregulation of three eicosanoids and two bile acids were associated with impaired kidney function, and these lipid species could be considered as alternative therapeutic targets to evaluate anti-fibrotic drugs and reveal undergoing biochemical mechanisms.

## 2. Results

### 2.1. Multivariate Analysis and Identification of Important Differential Lipid Species

**Figure 1:**
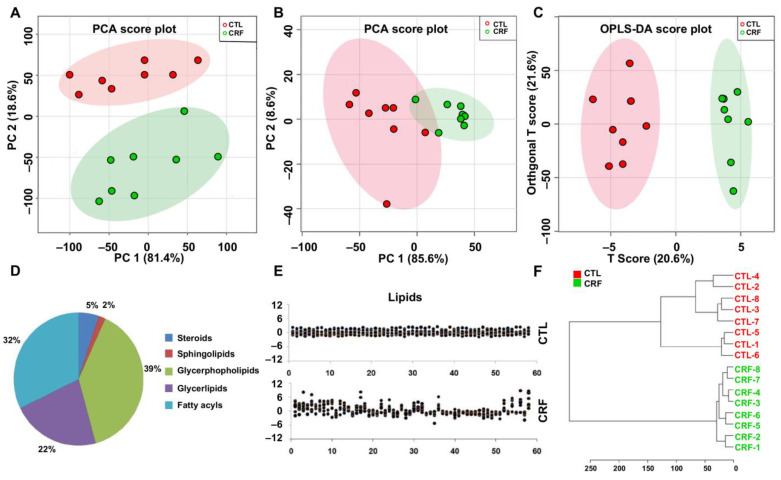
Altered lipid profiles were involved in chronic renal failure (CRF). (**A**) PCA plots with the scores of the first two principal components from CRF and control rats. (**B**) PCA plots with the scores of the first two principal components based on 58 lipid species from CRF and control rats. (**C**) OPLS-DA scores plots of the first two principal components based on 58 lipid species from CRF and control rats. (**D**) Pie chart presents the distribution of different classifications of 58 lipid species in CRF and control rats. (**E**) z-score plot of 58 altered lipid species in control and CRF rats. Each point represents an individual lipid in one sample. Z-score plots for the data are normalized to the mean of control samples. (**F**) Dendrogram of hierarchical clustering analysis based on 58 lipid species from CRF and control rats.

To analyze the systemic changes in lipid species in CRF and control rats, we performed an untargeted serum lipidomics using UPLC-HDMS. Multivariate statistical projection methods, including principal component analysis (PCA) and orthogonal partial least square-discriminant analysis (OPLS-DA), were employed for the analysis of metabolic profiles. According to UPLC-HDMS data, a total of 6606 m/z variables in positive ion mode were determined and processed by MarkerLynx XS. In the unsupervised PCA score plot, serum metabolic profiles from CRF and control rats were separated completely, indicating that lipid metabolic pattern was significantly changed in CRF rats (Figure 1A). Therefore, the altered metabolic profiling of serum lipids could reflect the changes in renal metabolism affected by adenine. In addition, xenobiotics and different fragment ions from the same lipids were excluded, 238 variables were chosen based on one-way ANOVA analysis (*p* < 0.05). Mann–Whitney U test and adjusted false discovery rate (FDR) (*p* < 0.05) were also applied to choosing differential lipids. In total, 58 lipids were identified in serum of CRF and control rats (Table 1 and Table S1). Here, we take m/z 393.2 in positive ion mode as an example to illustrate the identification methods. Besides ion at m/z 393.2, the ions at m/z 375.2403, 357.2407, and 339.2408 were also found (Table S1). Each of them has the m/z differences of H_2_O. Therefore, we speculated that three hydroxyl groups might be involved in the identified lipid. The 393.2 [M+H]^+^ might be a quasi-molecular ion. The ions at m/z 785.5 was found and it was speculated as [2M+H]^+^ ion. Collectively, the molecular weight of lipid was 392.2 Da. We further searched several databases, including Human Metabolome Database, Massbank, and Kyoto Encyclopedia of Genes and Genomes (KEGG), by using the molecular mass 392.2 Da; then, some compounds without three hydroxyl groups were removed from the candidate list. Taking all the information together, this ion was demonstrated to be chenodeoxycholic acid (CDCA). In addition, CDCA was confirmed by comparing this fragmentation pattern with a reference standard. By using the same method described above, potential lipids were identified (Table 1 and Table S1). These lipids were classified as the different types of lipids (Table 1). Among the 58 lipids, 27 lipids were significantly increased while 31 lipids were decreased in serum of CRF rats compared with control rats. Main lipid classes, including glycerophospholipids, glycerolipids, fatty acids, and steroids, were observed in four major clusters. Next, we further explored the impact of these selected lipids. First, PCA was performed to separate CRF rats from control rats. Data showed PCA of 58 differential lipids from CRF rats which could be separated from control rats (Figure 1B). As shown in Figure 1C, scattered points of various samples were classified into two clusters in OPLS-DA score plots, indicating that serum metabolism pattern significantly changed in CRF. The lipids mainly include 39% glycerophospholipids, 22% glycerolipids, 32% fatty acids, 5% sterols, and 2% sphingolipids (Figure 1D). The z-score showed that serum lipids are significantly altered in CRF rats (Figure 1E). Serum samples from CRF rats segregated into tight clusters at control rats (Figure 1F).

**Figure 2:**
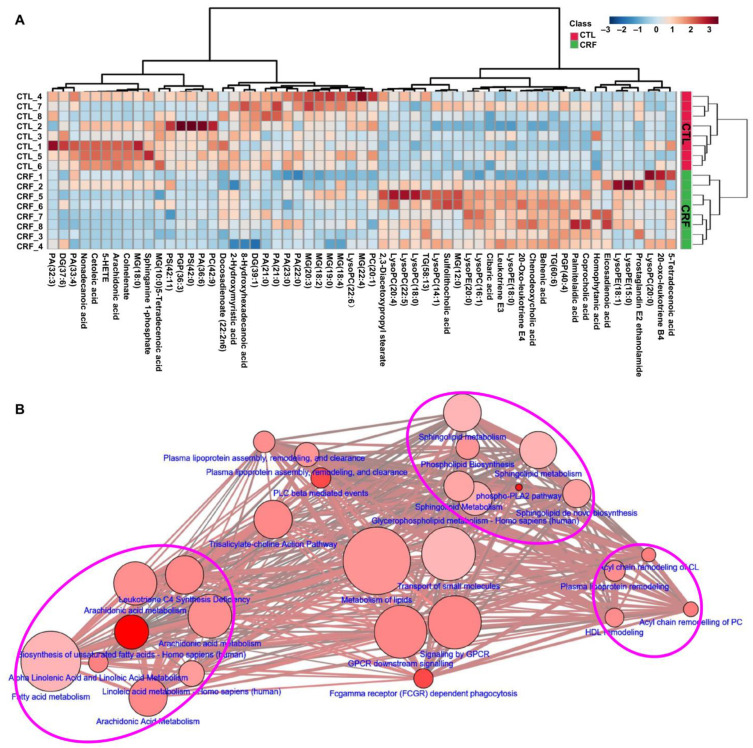
The dysregulation of lipid metabolic pathway participated in CRF. (**A**) Heatmap clustering analysis based on 58 lipid species from the CRF and control rats. (**B**) Metabolite pathway analysis of 58 lipid species by MetScape software running on cytoscape based on Kyoto Encyclopedia of Genes and Genomes (KEGG), Reactome, and SMPDB database. The node label color denotes the type of the metabolite sets. The size and color of each circle was based on pathway impact value and *p*-value, respectively. Node size indicates the amounts of metabolites found in metabolic pathway and more node size include the more amounts of metabolites in metabolic pathway. Node color indicates *p*-value. Red and pink indicate *p* < 0.0001 and *p* < 0.01, respectively.

To compare contribution of 58 altered lipids from CRF, we performed unsupervised cluster analyses focusing on each individual lipid species that exhibits statistically significant differences. Then heatmap analysis was performed to visualize the relative levels of the 58 differential lipids in each group (Figure 2A), and some lipids were significantly decreased while others were significantly increased in serum of the CRF rats. To further understand the function of the altered lipids, we mapped pathways overrepresented by identified lipid species in adenine-induced CRF and identified lipid metabolic networks by using various databases to determine the set that was most enriched by these lipid species. Figure 2B revealed that alterations of the 58 lipids were mainly involved in arachidonic acid (AA) metabolism, sphingolipid biosynthesis, and glycerophospholipid metabolism in CRF rats. In summary, these findings indicated that adenine leads to a significant lipid alteration in CRF, which is highly consistent with previous metabolome findings in CRF. These lipids could better reflect the metabolic changes of the renal failure.

### 2.2. Correlation Analyses between Important Differential Lipid Species and Serum Creatinine Levels

To further reveal differential lipids that might be associated with impaired kidney function, we explored the correlation analysis between each lipid levels and serum creatinine levels. The results showed that eight lipid species, including lysoPC(20:0), lysoPE(18:0), behenic acid, leukotriene E3, 20-Oxo-leukotriene E4, 20-Oxo-leukotriene B4, sulfolithocholic acid, and CDCA, have a strong correlation between each lipid and creatinine levels (R > 0.8512) (Table 1). The results demonstrated that the dysregulation of these lipid species was associated with impaired kidney function.

### 2.3. Predictive Performance Assessment

**Figure 3:**
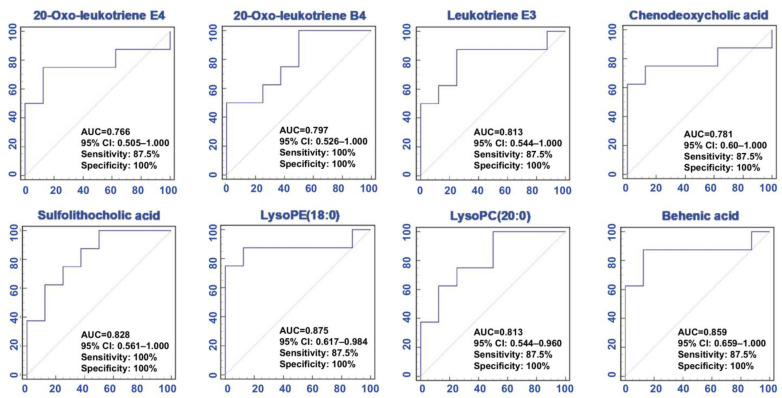
The levels of significantly altered lipid species linked with impaired kidney function in CRF. Eight lipid species were analyzed by using partial least square-discriminant analysis (PLS-DA)-based Receiver Operating Characteristic Curve (ROC) curves in CRF. The associated area under the curve (AUC), 95% confidence interval (CI), sensitivity, and specificity values were indicated.

To assess the predictive performance of the selected lipids, we performed Receiver Operating Characteristic Curve (ROC) analysis. The area under the curve (AUC), 95% confidence interval (95% CI), sensitivities, and specificities of 8 lipid species are shown in Figure 3. These lipid species have a high AUC value (>0.800), sensitivity (>80%) and specificity (>80%). The results suggested that the significant abnormalities of the lipid metabolic profiles and these specific lipid species could be considered to indicate renal failure caused by adenine.

### 2.4. Renoprotective Effect of PPU and ERG on Impaired Kidney Function

**Figure 4:**
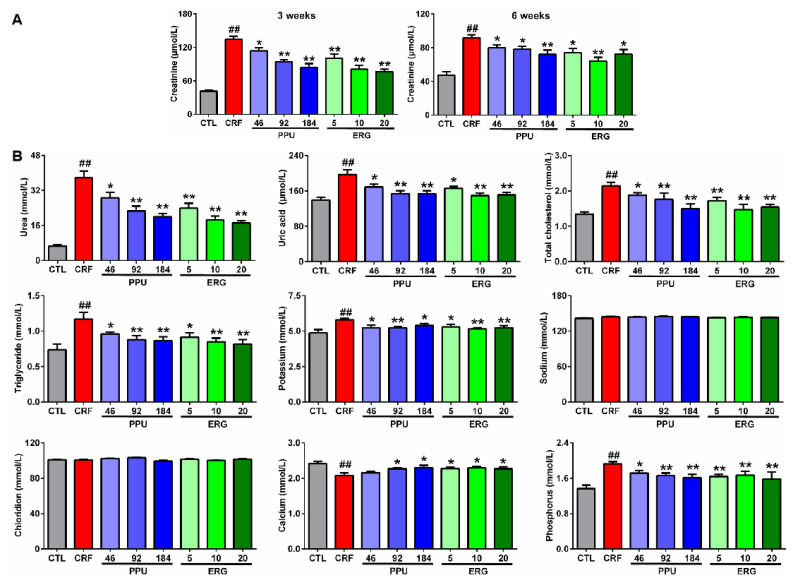
Treatment with Polyporus umbellatus (PPU) and ergone (ERG) improved impaired kidney function on adenine-induced CRF rats. (**A**) Serum creatinine levels in control, CRF, CRF+PPU, and CRF+ERG rats. (**B**) The levels of serum urea, uric acid, cholesterol, triglyceride, potassium, sodium, chloridion, calcium, and phosphorus in control, CRF, CRF+PPU, and CRF+ERG rats. ^##^—*p* < 0.01 compared with CTL (control) rats; *—*p* < 0.05, **—*p* < 0.01 compared with CRF rats.

The understanding of disease molecular mechanisms aims to discover novel therapeutic agents and strategies. In order to further demonstrate whether the restoration of the levels of eight lipid species, including eicosanoids and bile acids, were associated with improvement of kidney function, we further chose PPU (a renoprotective medicinal fungus) and its bioactive component ergone to assess their effects on eight lipids in adenine-induced CRF rats. As shown in Figure 4A, serum creatinine levels were significantly increased in rats induced by adenine compared with control rats at weeks 3 and 6. These results demonstrated that CRF rats showed declining renal function. Interestingly, the increasing trend of serum creatinine levels was obviously inhibited by the treatment with PPU and ERG (Figure 4A). Strikingly, both PPU and ERG treatments showed stronger inhibitory effects at week 3 compared with 6 weeks. Therefore, PPU and ERG treatments on CRF rats at week 3 were chosen to follow further research. PPU treatment caused a significantly decreased serum creatinine levels in a dose-dependent effect. Therefore, 184 mg/kg PPU was chosen as the optimal dose for further experiments. Similarly, the ERG of 10 mg/kg dose showed a strong inhibitory effect compared with ERG of 5 mg/kg dose, whereas the inhibitory effect of 20 mg/kg dose was similar to the inhibitory effect of 10 mg/kg dose. Therefore, the ERG of 10 mg/kg dose was chosen as the optimal dose for further experiments.

Data of other clinical biochemical parameters are shown in Figure 4B. Among these groups, compared with control rats, the CRF rats showed a greatly increasing trend in serum levels of urea, uric acid, cholesterol, triglyceride, potassium and phosphorus, whereas all these increases were improved by treatment both with PPU and ERG treatment. The level of calcium was significantly decreased in CRF rats compared with control rats, and this decrease was improved by treatment both with PPU and ERG treatment. But the levels of serum sodium and chloride did not show a significantly changes. Similarly, these results were also exhibit that PPU of 184 mg/kg dose and ERG of 10 mg/kg dose were the most optimal dose. Taken together, these results demonstrated that rats treated by adenine showed declining renal function. These results further confirmed that declining kidney function in CRF rats could be restored by PPU and ERG treatments.

### 2.5. Antifibrotic Effect of PPU and ERG

**Figure 5:**
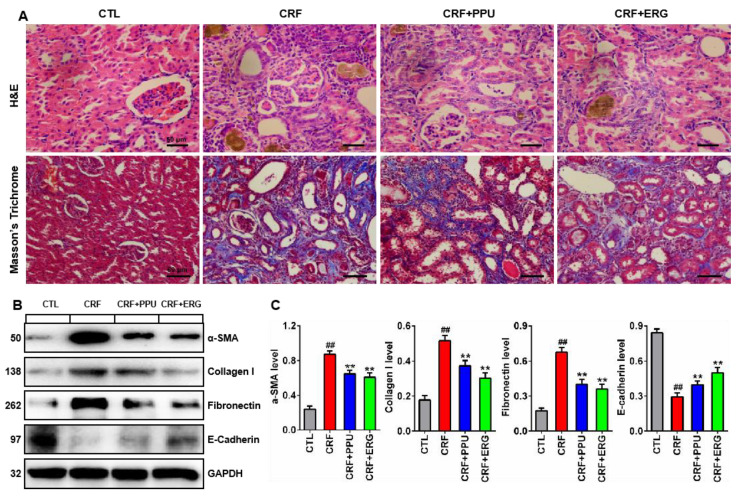
Treatment with PPU and ERG ameliorated renal fibrosis in adenine-induced CRF rats. (**A**) Histological findings of kidney tissues of hematoxylin & eosin (H&E) and Masson’s trichrome staining from control, CRF, CRF+PPU and CRF+ERG rats. H&E, scale bar, 50 μm; Masson’s trichrome staining, scale bar, 60 μm. (**B**) Protein expression of α-SMA, collagen I, Fibronectin, and E-cadherin in kidney tissues of control, CRF, CRF+PPU, and CRF+ERG rats. (**C**) Quantitative analysis of α-SMA, collagen I, Fibronectin, and E-cadherin in kidney tissues of control, CRF, CRF+PPU, and CRF+ERG rats. ^##^—*p* < 0.01 compared with CTL rats; ** *p* < 0.01 compared with CRF rats.

To further confirm the effect of PPU and ERG on renal fibrosis, hematoxylin & eosin (H&E) and Masson’s trichrome stainings were employed, as shown in Figure 5A. Morphologically, no significant abnormalities were observed in the kidney tissues in control rats. In contrast, CRF rats showed severe renal tubule-interstitial injury marked by tubular atrophy and dilation, epithelial denudation, sever inflammatory cell infiltration, granuloma formation in renal tissue stained with H&E, and marked renal interstitial fibrosis is observed by Masson’s trichrome staining. These results further sustained that the adenine-induced CRF model exhibited typical pathological features associated with CRF. In contrast, these pathological changes were improved in CRF rats treated by PPU and ERG. Moreover, Western blot analysis showed that adenine resulted in a significant increased protein expression of α-SMA, collagen I and fibronectin and decreased protein expression of E-cadherin, whereas treatment with PPU and ERG significantly reversed these profibrotic protein expressions compared with CRF rats (Figure 5B,C). Taken together, these results implied that both PPU and ERG attenuated renal fibrosis in adenine-induced CRF rats.

### 2.6. The Inhibitory Effect of PPU and ERG on the Levels of Eight Lipid Species

**Figure 6:**
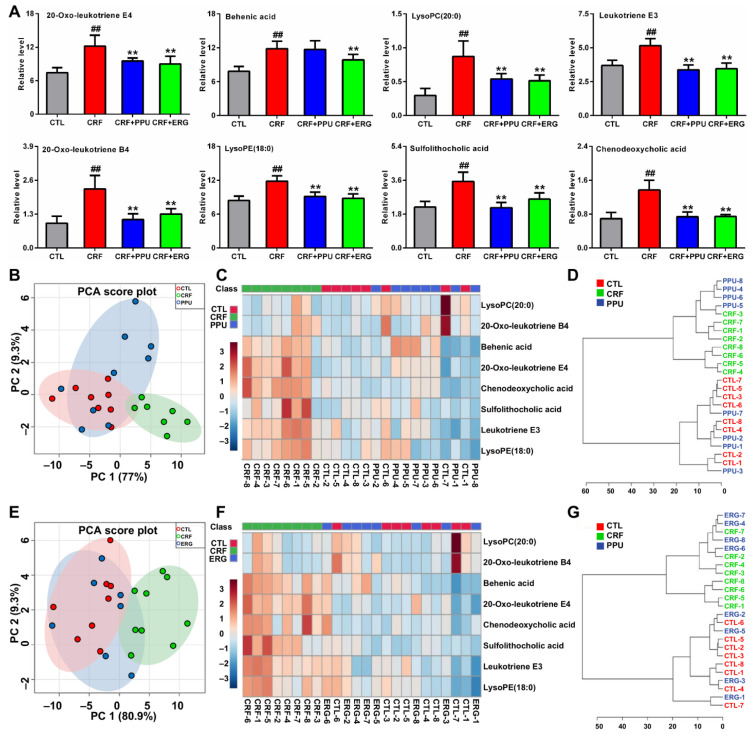
The dysregulation of eicosanoids and bile acids were restored by treatment of PPU and ERG. (**A**) Comparison of the relative intensities of eight lipid species in serum of control, CRF, CRF+PPU, and CRF+ERG rats. (**B**) PCA of two components of eight lipid species from control, CRF, and CRF+PPU rats. (**C**) Heatmap clustering analysis of eight lipid species in serum of control, CRF, and CRF+PPU rats. (**D**) Dendrogram of hierarchical clustering analysis based on eight lipid species in serum of control, CRF, and CRF+PPU rats. (**E**) PCA scores plot of eight lipid species from control, CRF, and CRF+ERG rats. (**F**) Heatmap clustering analysis of eight lipid species in serum of control, CRF, and CRF+ERG rats. (**G**) Dendrogram of hierarchical clustering analysis based on eight lipid species in serum of control, CRF, and CRF+ERG rats. ^##^—*p* < 0.01 compared with CTL rats; **—*p* < 0.01 compared with CRF rats.

In order to evaluate whether treatment with PPU and ERG have an effect on the levels of eicosanoids and bile acids in adenine-induced CRF rats, the levels of eight lipid species were determined in adenine-induced CRF rats treated by PPU and ERG. The results showed that eight lipid species were significantly increased in CRF rats compared with control rats, whereas the changes were completely restored in both PPU- and ERG-treated rats compared with CRF rats (Figure 6A). In PCA score plots, eight lipid species showed a satisfactory separation capacity among the three groups (Figure 6B). The PCA showed that adenine-induced CRF rats can be completely separated from control and PPU-treated rats, whereas PPU-treated rats cannot be separated from the control rats, which was consistent with the results of the clustering analysis (Figure 6C,D). Similarly, ERG was consistent with the results of PPU according to PCA scores plots and clustering analysis (Figure 6E–G). Collectively, these results demonstrated that these lipids could be used for assessing the drug intervention effect and revealing its biochemistry mechanism in CRF.

## 3. Discussion

Identification of lipid disorders and physiopathological mechanisms in the disease are critical steps for prevention and treatment of CRF. Structurally, lipids were divided into eight categories, including fatty acids, sphingolipids, glycerolipids, glycerophospholipids, saccharolipids, prenol lipids, sterol lipids, and polyketides [14]. In present study, we eventually identified a total of 58 differential lipid species which showed significant differences in CRF rats compared with control rats, and these lipids associated with the dysregulation of glycerophospholipid, glycerolipid, and fatty acid, as well as bile acid biosynthesis. Our findings demonstrated that eight lipids, including eicosanoids and bile acids, presented a strong linear correlation with serum creatinine levels. In addition, ROC analysis showed that eight lipids exhibited excellent AUC for differentiating CRF from control rats, with high sensitivity and specificity. Our findings demonstrated that the dysregulation of three eicosanoids and two bile acids were associated with impaired kidney function and they could indicate impaired kidney function in CRF. Treatment with PPU and ERG improved declining kidney function and protected against renal fibrotic effect in adenine-induced CRF rats. These altered lipids could be normalized or partially reversed by the treatment with PPU and ERG. Our findings further demonstrated that these eicosanoids and bile acids could be considered as alternative therapeutic targets to discover and evaluate anti-fibrotic drugs and reveal undergoing biochemical mechanisms.

Among identified lipid species, fatty acids accounted for most amounts of lipid species in the current study. Free fatty acids were produced by hydrolyzing the *sn*-2 acyl bond of glycerophospholipids via phospholipase A2 enzymes [39]. This study showed the increased levels of several saturated free fatty acids (FFA), including behenic acid, in adenine-induced rats compared with control rats. The redundant of FFA and saturated FFA are linked to higher risk of cardiovascular disease in patients with CKD [40,41]. The earlier studies have demonstrated that the levels of FFA and saturated fatty acids obviously were increased in plasma of patients with end-stage renal disease [40,42]. Wang et al. has reported that the levels of total FFA and saturated FFA and the values of FFA(16:1)/FFA(16:0) and FFA(18:1)/FFA(18:0) were significantly increased in patients with pre-hemodialysis than in healthy controls, whereas they were significantly decreased in patients after hemodialysis [43]. Friedman et al. has reported that high levels of serum saturated fatty acids positively correlated with sudden cardiac death in continuous hemodialysis patients [41]. Consistent with this conclusion is the findings that the levels of saturated FFA were demonstrated in adenine-induced rats. Mechanistically, FFA, such as palmitic acid, could induce the cell apoptosis by the upregulation of the expression of Bax and cleaved-caspase-3, as well as downregulation of the expression of Bcl-2 and phosphorylation of Nrf2, in proximal renal tubular epithelial (HK-2) cells [44]. In addition, Ibraheem et al. have demonstrated high saturated FFA has an important effect on renal failure progression in rats fed by high saturated FFA [45].

In contrast, the latest study showed that serum FFA levels were found to be associated with blood urea nitrogen levels. The FFA levels were significantly decreased in patients with higher blood urea nitrogen levels and lower estimated glomerular filtration rate [46]. The latest study by Rinschen et al. has demonstrated significantly decrease in FFA, including stearic acid, oleic acid, and linoleic acid, in glomeruli from salt-sensitive hypertensive rats [47]. Collectively, the current work, and that of others, suggests that significantly saturated FFA levels are implicated in CKD, and they contribute to declining kidney function and renal fibrosis.

Actually, glycerophospholipids were hydrolyzed to free fatty acids-mostly polyunsaturated fatty acids by phospholipase A2 enzymes. Linoleic acid and α-linolenic acid could convert into AA and eicosapentaenoic acid, respectively, and then AA and eicosapentaenoic acid could be metabolized into many classes of eicosanoids through cyclooxygenase, lipoxygenase and cytochrome P450 [48]. In the fatty acyl class, our current study further showed significantly increased levels of polyunsaturated fatty acids, especially eicosanoid metabolites, including 20-Oxo-leukotriene E4, leukotriene E3, and 20-Oxo-leukotriene B4, in adenine-induced CRF rats. Three eicosanoids present a high correlation with serum creatinine levels, indicating that they could mirror impaired kidney function in CRF. Wang et al. has reported that total eicosanoid levels were significantly increased in patients with pre-hemodialysis than in healthy controls, whereas they were significantly decreased in patients after hemodialysis [43]. Huang et al. found the increase in partial precursors of eicosanoids that were associated with increased inflammation symptom in uremic patients [49]. The latest study by Rinschen et al. has demonstrated significantly increase in 5-hydroxyeicosatetraenoic acid (5-HETE), 9-HETE, and 15-HETE in glomeruli, but not in tubules from salt-sensitive hypertensive rats [47]. In addition, the n-3 polyunsaturated fatty acids dietary supplementation significantly reduced release of leukotriene B4 and 5-HETE and significantly increased release of less inflammatory leukotriene B5 and 5-hydroxyeicosapentaenoic acid from stimulated neutrophil granulocytes in patients with CKD stage 2–5 compared with controls, whereas proteinuria levels and creatinine clearance did not improve [50]. PPU is a fungus used as a diuretic medicine, which possessed a variety of bioactivities, such as anti-inflammatory, antioxidative, anticancer, and antifibrotic effects [33]. Our earlier studies have demonstrated that ERG intervention retarded kidney function decline and renal fibrosis in aristolochic acid-induced nephropathy and adenine-induced CRF rats [37,38]. Treatment with PPU and ERG improved declining kidney function and protected against renal fibrotic effect in adenine-induced CRF rats. These altered lipids treated by PPU and ERG might attribute to the improvement of kidney function decline and renal fibrosis in adenine-induced CRF rats, in other words, PPU and ERG treatment first might ameliorate renal fibrosis and then improve the dysregulation of lipid metabolism. Taken together, the present study and others suggest that increased eicosanoid levels correlate with declining renal function. The differentially regulated eicosanoids 20-Oxo-leukotriene E4, leukotriene E3, and 20-Oxo-leukotriene B4 hold the promise to reveal novel targets to prevent CKD in the clinical setting.

One possible mechanism could be the impairment of fatty acid oxidation in the tubular epithelial cells from animal models and humans with interstitial fibrosis [27]. The previous study revealed significantly decreased expression of key enzymes and regulators of fatty acid oxidation and higher intracellular lipid deposition in humans and mouse models with tubulointerstitial fibrosis compared with the controls. Carnitine palmitoyltransferase 1 is an important rate-limiting enzyme in fatty acid oxidation. The expression of transcription factors, including peroxisome proliferator-activated receptors and peroxisome proliferator-activated receptor-γ coactivator-1a, were involved in fatty acid uptake and oxidation [51,52]. The metabolites of eicosanoids were primarily derived from AA, which could be metabolized into many classes of eicosanoids through cyclooxygenase, lipoxygenase and cytochrome P450 [48]. Patients with CKD progression could be predicted through increased levels of serum AA metabolites produced by lipoxygenase and cytochrome P450 [53]. Increased levels of serum AA metabolites, such as 15-HETE and 20-HETE, produced by alteration in cytochrome P450 family 4 activity has also been linked to CKD progression and hypertension [53]. In mice, several earlier publications have demonstrated that increased levels of AA metabolites via 5-lipoxygenase were involved in renal blood flow, albuminuria, tubulointerstitial injury and declining glomerular filtration rate [54,55]. Similar findings revealed the activities of 12-lipoxygenase and 15-lipoxygenase in mesangial expansion and albuminuria [56]. These mechanisms might explain increased levels of saturated FFA and eicosanoids in CKD. Furthermore, these mechanisms might be associated with improving aberrant metabolism of saturated FFA and eicosanoid by PPU and ERG treatment.

Another important finding was that two bile acids, including sulfolithocholic acid and CDCA, were demonstrated to be associated with impaired kidney function in CRF. Bile acids are important lipids of intestinal microbiota. Bile acids were initially synthesized from conjugation of cholesterol via hepatic enzymes in liver which play an important role in the digestion of dietary lipids [57]. Previous study has demonstrated that serum bile acid levels were elevated in parallel with the decreased estimated glomerular filtration rate in patients with CRF, while decreased bile acids in urine was observed in patients with CRF [58]. Li et al. has indicated that the profiles of altered serum bile acids, including CDCA, deoxycholic acid, and cholic acid, were significantly associated with dyslipidemia in patients with end-stage renal disease [59]. In addition, Wang et al. has reported that increased serum bile acid levels attributed to decreased bile acid filtration through kidneys in both patients and animal models with CRF [43]. Our earlier study has demonstrated the aberrant levels of serum bile acids, including CDCA, cholic acid, taurochenodesoxycholic acid, and 7-ketodeoxycholic acid, were observed in the different stages of rats with chronic aristolochic acid nephropathy; however, the levels of increased CDCA and cholic acid in serum were found in the early stages of aristolochic acid nephropathy, which might be used as biomarkers for prediction of early renal damage [25]. In contrast, it is well documented that bile acids in regulation of oxidative, inflammatory and fibrotic pathways [60]. Earlier study indicated that exogenous CDCA supplementation modulated renal lipid metabolism, inhibited inflammation and oxidative stress, reduced proteinuria and improved renal fibrosis [61]. These findings suggested that the kidneys play an important role in bile acid homeostasis. Our findings demonstrated that the levels of two bile acids could indicate impaired kidney function in CRF. However, treatment with PPU and ergone could restore the levels of two bile acids in the adenine-induced CRF rats. Therefore, targeting bile acids might be an alternative therapeutic strategy to improve impaired kidney function and retard renal fibrosis.

Copious studies have demonstrated abnormal glycerophospholipids metabolism contributes to the progression of renal disease in patients and animals with CRF from our work and others [22,62,63]. Our current results showed that two lysoglycerophosphocholines, including lysoPC(20:0) and lysoPE(18:0), were associated with impaired kidney function in CRF. Previous study has shown that increased lysoPC levels were positively correlated with elevated phospholipase A2 activity in patients with diabetic nephropathy [24]. Mechanistically, activated phospholipase A2 hydrolyses PC to produce lysoPC by catalyzing removal of fatty acid from the *sn*-2 position of PC. Increased lysoPC(20:0) and lysoPE(18:0) levels could be reversed in CRF rats treated by PPU and ERG. These mechanisms might explain improving aberrant metabolism of bile acids and lysophospholipids by PPU and ERG treatment.

There are some limitations to the study. In this study, some features in the mass spectra remain unidentified, and this used technology cannot distinguish some individual lipid species within isomeric clusters. The positive ion mode was used to determine the samples and identify the lipid species. We did not identify phosphatidylinositols in the current study. The negative ion mode might be performed for the identification of lipid species. In addition, the findings should be verified and addressed by using CKD patients.

In summary, exogenous adenine caused significantly changes in serum lipid species that were involved in impaired kidney function and renal fibrosis in CRF. Eight serum lipid species were correlated with serum creatinine levels. Intriguingly, the levels of three eicosanoids and two bile acids were associated with impaired kidney function, and they could indicate impaired kidney function in CRF. Treatment with PPU and ERG improved declining kidney function and protected against renal fibrosis. These eicosanoids and bile acids could be normalized or partially reversed by the treatment with PPU and ERG. Therefore, these eicosanoids and bile acids could be considered as alternative therapeutic targets to discover and evaluate anti-fibrotic drugs and reveal undergoing biochemical mechanisms.

## 4. Materials and Methods

### 4.1. Chemicals and Reagents

The extraction and isolation of PPU extract and ERG have been shown in the reported literature [64]. Adenine (batch No.: A8626, Purity 99.0%) and formic acid solution (ref BCBB6918, purity 50%), UPLC grade, were obtained from Sigma Chemical Co (Sigma Corp., St. Louis, MO, USA). Liquid-chromatography-grade acetonitrile and methanol were obtained from the Baker Company (Mallinckrodt Baker Inc., Phillipsburg, NJ, USA). Ultra-high purity water was prepared using a Milli-Q water purification system (Millipore Corp., Billerica, MA, USA). All other reagents were analytical grade and their purity was above 99.5%.

### 4.2. Animals Experiment and Sample Collection

All procedures involving animals were carried out according to the Guide for the Care and Use of Laboratory Animals of the State Committee of Science and Technology of the People’s Republic of China. The protocol was approved by Northwest University institutional animal care and use committee (Permit Number: SYXK 2010-004). Male SD rats were obtained from the Central Animal Breeding House of Xi’an Jiaotong University (Xi’an, China). Adenine-induced CRF model was reproduced as described in detail previously [21]. Male Sprague-Dawley rats initially weighing 190–210 g were maintained free access to water and under standardized conditions with a standard rodent diet and housed at a humidity-controlled room (ca. 60%) and temperature (ca. 23 °C) with a light/dark cycle of 12 h. CRF rats were treated with PPU extract and ergone or uremic clearance granule. Rats were randomly divided into control group, untreated CRF group, PPU-treated CRF group (CRF+PPU) and ergone-treated CRF group (CRF+ERG) (*n* = 8/group). In this study, the adenine was used for producing a rat model of CRF. CRF and different drug-treated CRF groups were given 200 mg/kg body weight of adenine dissolved in 1% (w/v) gum acacia solution by oral gavage once every day for 3 continuous weeks, which produced experimental renal failure. The healthy control group was treated with the vehicle alone throughout the experimental period. The PPU (46, 92, 184 mg/kg BW) and ERG (5, 10, 20 mg/kg BW) were given to CRF rats 3 h after each adenine dose by oral gavage once every day, respectively. At weeks 3 and 6, eight rats from each group were selected randomly and serum samples were collected. The rats were deeply anesthetized with sodium pentobarbital (60 mg/kg i.p.). The blood samples were obtained and centrifuged at 3000 rpm for 10 min to isolate the serum supernatant then stored at −80 °C for further analysis. The study was approved by the Ethical Committee of Northwest University, and all procedures were conducted in accordance with the Helsinki Declaration.

### 4.3. Renal Function Evaluation

Serum biochemistry were analyzed as described in detail previously [7]. The serum creatinine concentrations were determined to evaluate renal function [7,22] and the therapeutic efficacy of ergone and PPU in different time points. Biochemical parameters were measured using an Olympus AU 640 automatic analyzer (FV1000, Olympus, Tokyo Japan).

### 4.4. Histological Analysis and Western Blot Analysis

For histological analysis, kidney tissue was dissected out according to anatomical landmarks, weighed, and fixed in 4% paraformaldehyde and then embedded by using paraffin. Paraffin-embedded rat kidney sections (5-μm thickness) were prepared as a routine procedure. The sections were stained with Hematoxylin & eosin (H&E), Masson’s trichrome staining by standard protocol [32,65,66]. The morphological structure of each tissue sample was observed under a light microscope, and photomicrographs were taken. All the Western blot analysis were performed as described in detail previously [67,68]. The blots were obtained using Enhanced Chemiluminescence Reagents following a procedure of the manufacturer (Amersham Pharmacia Biotech, New Jersey, USA) and specific bands indicating target proteins were analyzed using Image J software. Glyceraldehyde-3-phosphate dehydrogenase (GAPDH) served as the internal control.

### 4.5. Metabolomic Analysis

Serum lipids were performed using an untargeted lipidomics UPLC-HDMS. The lipid profile procedure, including sample preparation, chromatographic separation and mass spectrometry methods, lipid separation and detection, data pre-processing, and statistical analysis for lipid identification, were performed following our previous protocols with minor modifications [22,69].

### 4.6. Linear Correlation Analysis and Receiver Operating Characteristic Curve (ROC) Analysis

Linear correlation analysis was performed between identified lipid species intensities and creatinine levels in the serum of CRF rats to choose lipid that were associated with impaired kidney function. In addition, partial least square-discriminant analysis (PLS-DA)-based ROC curve was performed for assessing the predictive performance of the selected lipids by using SPSS.

### 4.7. Pattern Recognition Analysis

PCA and OPLS-DA were performed to discriminate prominent differences among different groups using the MS data. Fold change (FC) were calculated based on mean ratios for CRF/control groups. Class-specific lipidomic pattern was visualized using heatmap with MetaboAnalyst software (version 4.0) and z-score plots analyses with R software (version 2.15.0). A metabolic network of identified lipids was constructed using integrated interaction databases.

### 4.8. Statistics Analysis

All statistical analyses were accomplished using the software in GraphPad Prism v 6.0 (San Diego, CA, USA) and SPSS statistical software version 25 (Chicago, IL, USA). The number of replicates was 8 per group for each data set, and the results were expressed as the mean ± SEM unless stated otherwise. Statistical comparisons between two groups were analyzed by either unpaired two-tailed Student’s t-test (normally distributed data) or two-tailed Mann–Whitney U test (non-normally distributed data) with a threshold of *p* < 0.05 in the SPSS 25 software. The resultant *p* values from ANOVA were further adjusted by a false discovery rate (FDR) based on the Hochberg-Benjamini method *p* < 0.05 indicated statistical significance.

## Figures and Tables

**Table 1:** Identification of significantly differential endogenous lipids in the rat serum.

Lipids	FC ^a^	*p* ^b^	*p* ^c^	FDR ^d^	AUC	R	Formula	Class
20-Oxo-leukotriene E4	1.642	4.23 × 10^−^^2^	7.40 × 10^−^^2^	4.72 × 10^−^^2^	0.77	0.942	C_23_H_35_NO_6_S	Fatty acids
Behenic acid	1.510	2.42 × 10^−^^2^	8.59 × 10^−^^1^	4.69 × 10^−^^2^	0.01	0.920	C_22_H_44_O_2_	Fatty acids
LysoPC (20:0)	2.517	3.82 × 10^−^^2^	3.50 × 10^−^^2^	4.27 × 10^−^^2^	0.81	0.897	C_28_H_58_NO_7_P	GPs
Leukotriene E3	1.396	4.06 × 10^−^^2^	3.60 × 10^−^^2^	5.02 × 10^−^^2^	0.81	0.885	C_23_H_39_NO_5_S	Fatty acids
20-Oxo- leukotriene B4	2.031	4.22 × 10^−^^2^	4.60 × 10^−^^2^	4.80 × 10^−^^2^	0.80	0.871	C_20_H_30_O_5_	Fatty acids
LysoPE (18:0)	1.405	1.67 × 10^−^^2^	1.20 × 10^−^^2^	5.39 × 10^−^^2^	0.88	0.870	C_23_H_48_NO_7_P	GPs
Sulfolithocholic acid	1.626	3.38 × 10^−^^2^	2.70 × 10^−^^2^	5.29 × 10^−^^2^	0.83	0.862	C_24_H_40_O_6_S	Steroids
CDCA	1.858	2.55 × 10^−^^2^	4.60 × 10^−^^2^	4.77 × 10^−^^2^	0.80	0.851	C_24_H_40_O_4_	Steroids
TG(60:6)	2.930	5.51 × 10^−^^3^	3.50 × 10^−^^2^	4.99 × 10^−^^2^	0.81	0.800	C_63_H_110_O_6_	Glycerolipids
LPC(16:1)	1.741	4.12 × 10^−^^2^	5.90 × 10^−^^2^	4.98 × 10^−^^2^	0.78	0.773	C_24_H_48_NO_7_P	GPs
MG(18:2)	0.530	7.83 × 10^−^^3^	6.00 × 10^−^^3^	3.79 × 10^−^^2^	0.91	0.772	C_21_H_38_O_4_	Glycerolipids
LysoPE(20:0)	1.713	2.83 × 10^−^^2^	2.70 × 10^−^^2^	4.82 × 10^−^^2^	0.83	0.771	C_25_H_52_NO_7_P	GPs
LysoPC(14:1)	1.584	4.47 × 10^−^^2^	2.70 × 10^−^^2^	4.80 × 10^−^^2^	0.83	0.770	C_22_H_44_NO_7_P	GPs
Cibaric acid	2.121	9.41 × 10^−^^3^	1.10 × 10^−^^2^	4.20 × 10^−^^2^	0.88	0.744	C_18_H_28_O_5_	Fatty acids
MG(20:3)	0.453	5.24 × 10^−^^3^	2.00 × 10^−^^3^	1.01 × 10^−^^2^	0.95	0.717	C_23_H_40_O_4_	Glycerolipids
Palmitelaidic acid	2.135	3.86 × 10^−^^2^	3.00 × 10^−^^2^	5.09 × 10^−^^2^	0.82	0.694	C_16_H_30_O_2_	Fatty acids
LysoPC (20:4)	2.866	3.94 × 10^−^^2^	2.10 × 10^−^^2^	5.08 × 10^−^^2^	0.84	0.691	C_28_H_50_NO_7_P	GPs
2,3-DOPS	1.465	3.63 × 10^−^^2^	1.60 × 10^−^^2^	5.40 × 10^−^^2^	0.86	0.691	C_25_H_46_O_6_	Glycerolipids
PC(42:9)	0.499	4.70 × 10^−^^2^	9.20 × 10^−^^2^	4.79 × 10^−^^2^	0.75	0.670	C_50_H_82_NO_8_P	GPs
PA(33:4)	0.736	4.30 × 10^−^^2^	4.60 × 10^−^^2^	4.70 × 10^−^^2^	0.80	0.667	C_36_H_63_O_8_P	GPs
LysoPC(22:6)	0.518	1.21 × 10^−^^2^	1.20 × 10^−^^2^	4.69 × 10^−^^2^	0.88	0.666	C_30_H_50_NO_7_P	GPs
CPCA	2.237	4.13 × 10^−^^2^	5.90 × 10^−^^2^	4.88 × 10^−^^2^	0.78	0.663	C_27_H_46_O_5_	Steroids
PA(21:0)	0.510	3.40 × 10^−^^2^	2.10 × 10^−^^2^	5.20 × 10^−^^2^	0.84	0.622	C_24_H_47_O_8_P	GPs
LysoPC(18:0)	2.161	1.90 × 10^−^^2^	9.00 × 10^−^^3^	5.01 × 10^−^^2^	0.89	0.614	C_26_H_54_NO_7_P	GPs
MG(19:0)	0.513	2.19 × 10^−^^2^	1.60 × 10^−^^2^	4.89 × 10^−^^2^	0.86	0.601	C_22_H_44_O_4_	Glycerolipids
PC(20:1)	0.416	6.58 × 10^−^^3^	6.00 × 10^−^^3^	4.63 × 10^−^^2^	0.91	0.598	C_28_H_56_NO_7_P	GPs
Homophytanic acid	1.296	2.27 × 10^−^^2^	9.00 × 10^−^^3^	4.55 × 10^−^^2^	0.89	0.597	C_21_H_42_O_2_	Fatty acids
PA(22:0)	0.662	2.22 × 10^−^^2^	2.70 × 10^−^^2^	4.77 × 10^−^^2^	0.83	0.574	C_25_H_49_O_8_P	GPs
MG(22:4)	0.543	2.87 × 10^−^^2^	9.00 × 10^−^^3^	4.75 × 10^−^^2^	0.89	0.571	C_25_H_42_O_4_	Glycerolipids
TG(58:13)	3.66	2.26 × 10^−^^2^	1.10 × 10^−^^2^	4.69 × 10^−^^2^	0.88	0.514	C_61_H_92_O_6_	Glycerolipids
MG(12:0)	1.510	3.96 × 10^−^^2^	2.70 × 10^−^^2^	4.99 × 10^−^^2^	0.83	0.509	C_15_H_30_O_4_	Glycerolipids
5-TCA	2.883	3.85 × 10^−^^2^	4.30 × 10^−^^2^	4.19 × 10^−^^2^	0.80	0.449	C_14_H_26_O_2_	Fatty acids
PA(36:6)	0.156	2.27 × 10^−^^3^	2.00 × 10^−^^3^	1.32 × 10^−^^2^	0.95	0.402	C_39_H_65_O_8_P	GPs
PS(42:0)	0.269	3.17 × 10^−^^2^	5.00 × 10^−^^3^	5.01 × 10^−^^2^	0.92	0.359	C_48_H_94_O_10_P	GPs
Docosadienoate	0.601	2.09 × 10^−^^2^	3.60 × 10^−^^2^	4.85 × 10^−^^2^	0.81	0.322	C_22_H_40_O_2_	Fatty acids
LysoPC(22:5)	2.268	1.78 × 10^−^^2^	6.00 × 10^−^^3^	5.07 × 10^−^^2^	0.91	0.317	C_30_H_52_NO_7_P	GPs
Arachidonic acid	0.257	2.57 × 10^−^^2^	5.50 × 10^−^^2^	4.65 × 10^−^^2^	0.78	0.314	C_20_H_32_O_2_	Fatty acids
Nonadecanoic acid	0.267	7.02 × 10^−^^3^	9.00 × 10^−^^3^	5.09 × 10^−^^2^	0.89	0.311	C_19_H_38_O_2_	Fatty acids
PGP(36:3)	0.208	3.80 × 10^−^^2^	6.00 × 10^−^^3^	4.38 × 10^−^^2^	0.91	0.308	C_42_H_78_O_13_P_2_	GPs
PA(23:0)	0.676	1.92 × 10^−^^2^	2.10 × 10^−^^2^	4.84 × 10^−^^2^	0.84	0.304	C_26_H_51_O_8_P	GPs
DG(37:6)	0.379	1.72 × 10^−^^2^	4.40 × 10^−^^2^	5.00 × 10^−^^2^	0.80	0.301	C_40_H_66_O_5_	Glycerolipids
PA(32:3)	0.470	4.15 × 10^−^^2^	1.60 × 10^−^^2^	4.82 × 10^−^^2^	0.86	0.294	C_35_H_63_O_8_P	GPs
MG(18:0)	0.240	1.01 × 10^−^^2^	1.20 × 10^−^^2^	4.17 × 10^−^^2^	0.88	0.291	C_21_H_42_O_4_	Glycerolipids
Cetoleic acid	0.271	7.16 × 10^−^^3^	9.00 × 10^−^^3^	4.62 × 10^−^^2^	0.89	0.278	C_22_H_42_O_2_	Fatty acids
5-HETE	0.261	6.89 × 10^−^^3^	9.00 × 10^−^^3^	4.71 × 10^−^^2^	0.89	0.271	C_20_H_32_O_3_	Fatty acids
DG(39:1)	0.284	7.51 × 10^−^^3^	2.00 × 10^−^^2^	3.96 × 10^−^^2^	0.84	0.265	C_42_H_80_O_5_	Glycerolipids
MG(10:0)	0.571	2.82 × 10^−^^2^	2.10 × 10^−^^2^	4.95 × 10^−^^2^	0.84	0.253	C_13_H_26_O_4_	Glycerolipids
MG(18:4)	0.559	4.56 × 10^−^^2^	2.70 × 10^−^^2^	4.72 × 10^−^^2^	0.83	0.232	C_21_H_34_O_4_	Glycerolipids
Sphinganine 1-phosphate	0.447	2.64 × 10^−^^3^	5.00 × 10^−^^2^	4.65 × 10^−^^2^	0.92	0.214	C_18_H_40_NO_5_P	Sphingolipids
Eicosapentaenoic acid	0.264	6.76 × 10^−^^3^	9.00 × 10^−^^2^	4.54 × 10^−^^2^	0.89	0.174	C_22_H_42_O_2_	Fatty acids
Hydroxymyristic acid	0.609	1.50 × 10^−^^2^	2.10 × 10^−^^2^	4.32 × 10^−^^2^	0.84	0.129	C_14_H_28_O_3_	Fatty acids
ProstaglandinE2	1.345	3.71 × 10^−^^2^	4.60 × 10^−^^2^	4.38 × 10^−^^2^	0.80	0.125	C_22_H_37_NO_5_	Fatty acids
Colnelenate	0.205	1.84 × 10^−^^2^	9.00 × 10^−^^3^	5.09 × 10^−^^2^	0.89	0.114	C_18_H_28_O_3_	Fatty acids
LysoPE(15:0)	4.687	4.96 × 10^−^^2^	2.00 × 10^−^^2^	4.96 × 10^−^^2^	0.84	0.107	C_20_H_42_NO_7_P	GPs
PS(42:11)	0.192	4.48 × 10^−^^2^	6.00 × 10^−^^3^	4.72 × 10^−^^2^	0.91	0.090	C_48_H_72_O_10_P	GPs
LysoPE(18:1)	6.434	2.07 × 10^−^^2^	4.30 × 10^−^^2^	5.01 × 10^−^^2^	0.80	0.075	C_23_H_46_NO_7_P	GPs
HOCA	0.713	1.45 × 10^−^^2^	1.20 × 10^−^^2^	4.25 × 10^−^^2^	0.88	0.042	C_16_H_32_O_3_	Fatty acids
Eicosadienoic acid	11.09	7.44 × 10^−^^3^	1.00 × 10^−^^3^	4.31 × 10^−^^2^	0.97	0.012	C_20_H_36_O_2_	Fatty acids

^a^—Fold change (FC) was obtained by comparing those lipids in CRF rats with the controls; FC with a value > 1 indicated a relatively higher intensity presenting in CRF, whereas a value < 1 indicated a relatively lower intensity compared with the controls. ^b^—The *p* value was calculated from Student’s t test. ^c^—The *p* value was calculated from nonparametric test Mann–Whitney U test. ^d^—Value of false discovery rate (FDR) was obtained from the adjusted *p*-value of FDR correction by Benjamini-Hochberg method. AUC, area under the curve; CDCA, chenodeoxycholic acid; GPs, glycerophospholipids.

## Data Availability

All data are available in the manuscript and in the supplementary material.

## Supplementary Materials

The following are available online at https://www.mdpi.com/2218-1989/11/2/127/s1, Table S1: The MS and MS/MS and fragment ions of identified lipids in the rat serum.
