# Supplementary material for: The Dysregulation of Eicosanoids and Bile Acids Correlates with Impaired Kidney Function and Renal Fibrosis in Chronic Renal Failure
Source: Metabolites. 2021 Feb 23;11(2):127. doi: 10.3390/metabo11020127 (PMC7926759; doi:10.3390/metabo11020127)
Supplement: Supplementary file 1 [file metabolites-11-00127-s001.pdf]

Table S1 The MS and MS/MS and fragment ions of identified lipids in the rat serum

t_R	Lipids	MS and MS/MS	Fragment ions
6.46	20-Oxo-leukotriene E4	454.2930; 436.2915; 418.2925	$[M+H]^+$; $[M+H-H_2O]^+$; $[M+H-2H_2O]^+$;
6.99	Behenic acid	341.3047; 323.3041	$[M+H]^+$; $[M+H-H_2O]^+$
6.84	LysoPC(20:0)	574.4030; 206.0349; 126.0281	$[M+Na]^+$; $[C_5H_{14}NO_4PNa]^+$; $[C_5H_{13}NONa]^+$
7.04	Leukotriene E3	464.3133; 442.2634; 424.2645	$[M+Na]^+$; $[M+H]^+$; $[M+H-H_2O]^+$
6.80	20-Oxo-leukotriene B4	723.5073; 351.2298; 333.2312	$[2M+Na]^+$; $[M+H]^+$; $[M+H-H_2O]^+$;
7.04	LysoPE(18:0)	482.3243; 464.3293; 141.0345	$[M+H]^+$; $[M+H-H_2O]^+$; $[C_2H_8NO_4P]^+$
6.63	Sulfolithocholic acid	439.3005; 421.3002	$[M+H]^+$; $[M+H-H_2O]^+$
6.46	Chenodeoxycholic acid	393.2400; 375.2403; 357.2407; 339.2408	$[M+H]^+$; $[M+H-H_2O]^+$; $[M+H-2H_2O]$+; $[M+H-3H_2O]^+$
7.05	TG(60:6)	963.6418	$[M+H]^+$
7.25	LysoPC(16:1)	511.3961; 201.0452; 121.0383	$[M+NH_4]^+$; $[C_5H_{18}N_2O_4P]^+$; $[C_5H_{17}N_2O]^+$
6.04	MG(18:2)	372.3101; 355.3145	$[M+NH_4]^+$; $[M+H]^+$
7.25	LysoPE(20:0)	510.3916; 492.3965; 141.0346	$[M+H]^+$; $[M+H-H_2O]^+$; $[C_2H_8NO_4P]^+$
7.21	LysoPC(14:1)	466.3293; 448.3343; 184.0451; 104.0382	$[M+H]^+$; $[M+H-H_2O]^+$; $[C_5H_{15}NO_4P]^+$; $[C_5H_{14}NO]^+$
6.99	Cibaric acid	342.3085; 307.3078	$[M+NH_4]^+$; $[C_{18}H_{27}O_4]^+$
6.16	MG(20:3)	398.3422	$[M+NH_4]^+$
7.21	Palmitelaidic acid	509.3810; 255.2375; 237.2385	$[2M+H]^+$; $[M+H]^+$; $[M+H-H_2O]^+$
5.35	LysoPC(20:4)	544.2665; 526.2715; 184.0450; 104.0381	$[M+H]^+$; $[M+H-H_2O]^+$; $[C_5H_{15}NO_4P]^+$; $[C_5H_{14}NO]^+$
6.75	2,3-Diacetoxypropyl stearate	465.3169; 443.3256	$[M+Ma]^+$; $[M+H]^+$
7.71	PC(42:9)	856.5841; 838.5839; 184.0451; 104.0382	$[M+H]^+$; $[M+H-H_2O]^+$; $[C_5H_{15}NO_4P]^+$; $[C_5H_{14}NO]^+$
7.28	PA(33:4)	655.3351;637.3348	$[M+H]^+$; $[M+H-H_2O]^+$
6.19	LysoPC(22:6）	590.3215; 206.0350; 126.0282	$[M+Na]^+$; $[C_5H_{14}NO_4PNa]^+$; $[C_5H_{13}NONa]^+$
6.77	Coprocholic acid	468.3070; 451.3130; 433.3131; 415.3229	$[M+NH_4]^+$; $[M+H]^+$; $[M+H-H_2O]^+$; $[M+H-2H_2O]^+$
6.05	PA(21:0)	495.3274; 477.3271	$[M+H]^+$; $[M+H-H_2O]^+$
5.42	LysoPC(18:0)	524.3141; 506.3191; 184.0452; 104.0383	$[M+H]^+$; $[M+H-H_2O]^+$; $[C_5H_{15}NO_4P]^+$; $[C_5H_{14}NO]^+$
6.04	MG(19:0)	373.3142	$[M+H]^+$
7.14	PC(18:1(9Z)e/2:0)	572.3694; 206.0349; 126.0281	$[M+Na]^+$; $[C_5H_{14}NO_4PNa]^+$; $[C_5H_{13}NONa]^+$
6.43	Homophytanic acid	344.3118; 309.3124	$[M+NH_4]^+$; $[C_{21}H_{41}O]^+$
6.22	PA(22:0)	547.3493; 509.3247	$[M+K]^+$; $[M+H]^+$
6.27	MG(22:4)	424.3424; 407.3428	$[M+NH_4]^+$; $[M+H]^+$

6.62	TG(58:13)	959.6471	$[M+K]^+$
6.75	MG(12:0)	292.2996; 275.2789	$[M+NH_4]^+$; $[M+H]^+$
4.70	5-Tetradecenoic acid	244.2059; 209.3014	$[M+NH_4]^+$; $[C_{14}H_{25}O]^+$
4.44	PA(36:6)	731.3811; 693.4473	$[M+K]^+$; $[M+H]^+$
4.44	PS(42:0)	914.5480; 876.5989	$[M+K]^+$; $[M+H]^+$
5.84	Docosadienoate (22:2n6)	319.3034	$[M+H-H_2O]^+$
5.73	LysoPC(22:5)	570.3555; 552.3605; 184.0451; 104.0382	$[M+H]^+$; $[M+H-H_2O]^+$; $[C_5H_{15}NO_4P]^+$; $[C_5H_{14}NO]^+$
8.18	Arachidonic acid	322.3189; 287.3178	$[M+NH_4]^+$; $[C_{20}H_{31}O]^+$
8.18	Nonadecanoic acid	321.3153; 299.3547; 281.3541	$[M+Na]^+$; $[M+H]^+$; $[M+H-H_2O]^+$
4.44	PGP(36:3)	853.4714; 835.4714; 755.4774	$[M+H]^+$; $[M+H-H_2O]^+$; $[M+H-H_3PO_4]^+$
6.24	PA(23:0)	523.3492; 505.3487	$[M+H]^+$; $[M+H-H_2O]^+$
6.40	DG(37:6)	627.5356	$[M+H]^+$
7.28	PA(32:3)	681.3508; 643.4321	$[M+K]^+$; $[M+H]^+$
7.43	MG(18:0)	376.3185; 359.3175	$[M+NH_4]^+$; $[M+H]^+$
8.18	Cetoleic acid	339.3450; 321.3459	$[M+H]^+$; $[M+H-H_2O]^+$
8.19	5-HETE	338.3419; 303.3414	$[M+NH_4]^+$; $[C_{20}H_{31}O_3]^+$
7.70	DG(39:1)	703.5730	$[M+K]^+$
5.85	MG(10:0)	247.2423	$[M+H]^+$
5.49	MG(18:4)	368.2773; 351.2874	$[M+NH_4]^+$; $[M+H]^+$
5.98	Sphinganine 1-phosphate	382.2715; 364.2714	$[M+H]^+$; $[M+H-H_2O]^+$
8.18	Eicosapentaenoic acid	303.3044; 285.2594	$[M+H]^+$; $[M+H-H_2O]^+$
4.76	Hydroxymyristic acid	262.2380; 227.2394	$[M+NH_4]^+$; $[C_{14}H_{27}O_2]^+$
6.29	Prostaglandin E2 ethanolamide	396.3469	$[M+H]^+$
7.80	Colnelenate	310.3105; 275.4133	$[M+NH_4]^+$; $[C_{18}H_{27}O_2]^+$
4.40	LysoPE(15:0)	462.2676; 163.0458	$[M+Na]^+$; $[C_2H_7NO_4PNa]^+$
4.44	PS(42:11)	854.4743; 836.4728	$[M+H]^+$; $[M+H-H_2O]^+$
3.91	LysoPE(18:1)	480.2781; 462.2833; 141.0343	$[M+H]^+$; $[M+H-H_2O]^+$; $[C_2H_8NO_4P]^+$
5.34	Hydroxyhexadecanoic acid	290.2691; 273.2758	$[M+NH_4]^+$; $[C_{16}H_{31}O_2]^+$
5.67	Eicosadienoic acid	347.2219; 309.3258	$[M+K]^+$; $[M+H]^+$
